# High-intensity exercise prescription guided by heart rate variability in breast cancer patients: a study protocol for a randomized controlled trial

**DOI:** 10.1186/s13102-023-00634-2

**Published:** 2023-03-08

**Authors:** Ana Myriam Lavín-Pérez, Daniel Collado-Mateo, Carmen Hinojo González, Ana de Juan Ferré, Cristina Ruisánchez Villar, Xián Mayo, Alfonso Jiménez

**Affiliations:** 1 Centre for Sport Studies, Rey Juan Carlos University, Madrid, Spain; 2 GO fitLAB, Ingesport, Madrid, Spain; 3 Program of Epidemiology and Public Health (Interuniversity), PhD International School of the Rey Juan Carlos University, Madrid, Spain; 4 Onchology Department, Hospital Universitario Marqués de Valdecilla and Instituto de Investigación Marqués de Valdecilla (IDIVAL)., Santander, Spain; 5 Cardiology Department, Hospital Universitario Marqués de Valdecilla and Instituto de Investigación Marqués de Valdecilla (IDIVAL)., Santander, Spain; 6 Advanced Wellbeing Research Centre, College of Health, Wellbeing and Life Sciences, Sheffield Hallam University, Sheffield, UK

**Keywords:** Breast cancer survivors, HIT, Strength training, Remote and supervised exercise, Cardiotoxicity, Physical effects, Psychosocial effects

## Abstract

**Background:**

Breast cancer is a chronic disease with a large growth in its treatments, prognosis, improvements, side effects and rehabilitation therapies research. These advances have also highlighted the need to use physical exercise as a countermeasure to reduce the cardiotoxicity of pharmacological treatments, increase patients' strength and quality of life and improve body composition, physical condition and mental health. However, new investigations show the need for a closed exercise individualisation to produce higher physiological, physical and psychological benefits in remote exercise programs. To this end, the present study will use, in a novel way in this population, heart rate variability (HRV) as a measure for prescribing high-intensity training. Thus, the primary objective of this randomised clinical trial is to analyse the effects of a high-intensity exercise program daily guided by HRV, a preplanned moderate to high-intensity exercise intervention and a usual care group, in breast cancer patients after chemotherapy and radiotherapy treatments.

**Methods:**

For this purpose, a 16-week intervention will be carried out with 90 breast cancer patients distributed in 3 groups (a control group, a moderate to high-intensity preplanned exercise group and a high-intensity exercise group guided by HRV). Both physical exercise interventions will be developed remotely and supervised including strength and cardiovascular exercises. Physiological variables, such as cardiotoxicity, biomarkers, lipid profile, glucose, heart rate and blood pressure; physical measures like cardiorespiratory capacity, strength, flexibility, agility, balance and body composition; and psychosocial variables, as health-related quality of life, fatigue, functionality, self-esteem, movement fear, physical exercise level, anxiety and depression will be measure before, after the intervention and 3 and 6 months follow up.

**Discussion:**

Personalized high-intensity exercise could be a promising exercise intervention in contrast to moderate-intensity or usual care in breast cancer patients to reach higher clinical, physical and mental effects. In addition, the novelty of controlling HRV measures daily may reflect exercise effects and patients' adaptation in the preplanned exercise group and a new opportunity to adjust intensity. Moreover, findings may support the effectiveness and security of physical exercise remotely supervised, although with high-intensity exercise, to reach cardiotoxicity improvements and increase physical and psychosocial variables after breast cancer treatments.

*Trial registration* ClinicalTrials.gov nº NCT05040867 (https://clinicaltrials.gov/ct2/show/record/NCT05040867).

**Supplementary Information:**

The online version contains supplementary material available at 10.1186/s13102-023-00634-2.

## Background

Worldwide cancer incidence is growing each year reaching 19.3 million new cancer cases in 2020. Whereas it is expected to worsen to 28.4 million cases in 2040, an increase of 47%. Concretely, female breast cancer has surpassed lung cancer as the most diagnosed type, with an estimated 2.3 million new cases every year (11.7%), accounting for 1 in 4 cancer cases and being the fifth leading cause of cancer mortality worldwide, with 685,000 yearly deaths. However, although new advances and treatments are increasing cancer-related survivorship, estimated to be 29.1% higher in 2029 [1], breast cancer survivors had an almost twofold higher risk of dying compared with cancer-free women [2]. An aspect to consider given that by 2040 it is expected that 73% of survivors will be over 65 years of age [1].

One of the principal causes of these differences leads to cancer and treatments side-effects in breast cancer survivors increasing their comorbidity rates and decreasing patients' health-related quality of life (HRQoL) and survivorship [3]. Regarding long-term side effects, breast cancer patients may suffer, even several years after the diagnosis, cardiotoxicity [4], autonomic dysfunction[5], sarcopenia [6, 7], joint pain and cancer-related fatigue [8], among others, are quite common and severe. These interrelated consequences, caused among other aspects by pharmacological toxicity [6, 9] and physical inactivity, are associated with mortality [10–12], worse prognosis and cancer evolution [13, 14] and morbidity [15]. Cardiotoxicity, the toxic effect produced in patients' cardiovascular system by some anticancer drugs, could cause the appearance of several cardiovascular abnormalities such as hypotension or hypertension, cardiorespiratory capacity decline, arrhythmias, myocardial infarction, thromboembolism, myocarditis, greater arterial rigidity, over-activation of the sympathetic nervous system and a parasympathetic decline -measured by heart rate variability (HRV)-[16–18]. Such is its gravity, that breast cancer survivors are at high risk of heart failure [19], autonomic dysfunction [20] and cardiovascular disease [19] up to the point of being the second most common cause of death among breast cancer survivors [21].

However, physical exercise programs, as recent literature is showing, could be an effective tool to reduce these severe side effects [22]. Although the investigations performed in breast cancer patients, not murine models, are limited it seems that exercise may increase left ventricular ejection fraction and cardiorespiratory capacity [23], decrease biomarkers such as Brain Natriuretic Peptide (BNP), high-sensitivity cardiac troponin and c-reactive protein [22, 24, 25] and blood pressure, and improve the sympathovagal imbalance increasing baroreflex sensibility and reducing resting heart rate [26, 27]. These physiological changes have been proved to be produced specially by endurance training due to DOX-induced increases in oxidative stress and apoptosis, decreases chronic inflammation, angiotensin II, renin and atherosclerosis and increases the patient's vasodilatation [25]. To reach the mentioned effects it seems that exercise programs focused on cardiotoxicity need to involve at least 36 sessions [24]. The exercise intensity carried out in the interventions is quite heterogeneous involving most of them from moderate to high intensity [22, 24, 25]. Whereas, more current research, but not focused on cardiotoxicity, has found also positive psychosocial, physical and physiological effects with high-intensity exercise interventions [23, 26] and it seems to be a potential tool to provide a higher glycolytic metabolism [28], induce a decrease of intratumorally lactate concentration [29] and moderate the overexpression of reactive oxygen species limiting the tumor growth or cancer recurrence and inflammation [30].

Nevertheless, most of the interventions focused their exercise programs only on aerobic training forgetting the role of muscle mass and function to reduce the toxicity produced by treatments and tumor prognosis [6], even though sarcopenia is associated with an increased risk of overall mortality in breast cancer survivors and breast-cancer-specific mortality [11, 31, 32]. In this regard, resistance training interventions have been stated to be crucial to recovering the loss of muscle mass and muscle function caused by chemotherapy [33, 34], reducing myomatosis and chronic inflammation [35], reducing oxidative free radicals and oxidative stress [35], improving body composition [36], reducing pain join and cancer rated fatigue [37], increasing cardiorespiratory fitness [23, 38] and HRQoL [37], reducing mortality from cardiovascular disease [39] and all causes of death [40]. Commonly, resistance programs, performed alone or like concurrent training, lasted at least 12 weeks, utilized a moderate intensity, between 50 and 80% of 1 repetition maximum (RM) or high intensity < 85% of 1 RM [36, 37].

The debate and the controversial results about optimal exercise intensity and type for breast cancer survivors could be produced due to cancer investigation and clinical practice, especially in group-based programs, all the participants from the same group train at the same corresponding intensity. In this regard, programs are not daily personalized without considering participants' daily physiological and psychological requirements. So that, those participants who do not respond correctly to high-intensity programs could be because they do not tolerate the pre-planned intensity equally every day. Innovative investigations in sports sciences are starting to prescribe daily training intensity by utilizing participants' matutine heart rate variability [39] a new useful tool not employed before, as far as we are concerned, in breast cancer survivors. HRV provides a multidimensional registration of autonomic modulation [40] presenting the physiological stress and the sympathovagal imbalance, so common in breast cancer survivors [5]. Besides, some investigations have stated the relation between pain perception, so common in breast cancer patients, and HRV scorings, showing that people with high pain have lower parasympathetic parameters [41]. For these reasons, the current study will aim to prescribe high-intensity exercise involving cardiovascular and resistance training, guided daily by HRV trying to individualize exercise to patients’ physiological stress.

Moreover, concerning the new habits and the current risk due to the coronavirus pandemic (COVID-19) there is a need for novel intervention in a supervised and remote-controlled context. In this sense, it could be also one of the first exercise programs where the physical specialist control and monitor each exercise session in real-time in contrast to most remote interventions based on self-delivery exercise, physical exercise recommendations or mobile apps [42].

### Aim and research questions

Therefore, given the potential benefits of physical exercise and the possibilities of optimizing how it is prescribed for cancer patients, the current randomized controlled trial aims to analyze the effectiveness of a high-intensity remote exercise program guided daily by HRV compared to a pre-planned remote moderate to high-intensity exercise program in breast cancer survivors. For this purpose, the effects of both physical exercise programs on physiological (cardiotoxicity, inflammatory factors, cardiovascular variables, body composition and anthropometric parameters), physical (low and upper body strength, cardiorespiratory fitness, low and upper body flexibility, agility and balance) and psychological health (HRQoL, fatigue, life satisfaction, self-esteem, anxiety and depression, shoulder disability perception, physical activity, kinesophobia and exercise motivation) measurements of breast cancer patients will be assessed and compared an inactive control group.

## Methods/design

### Study design

**Fig. 1:**
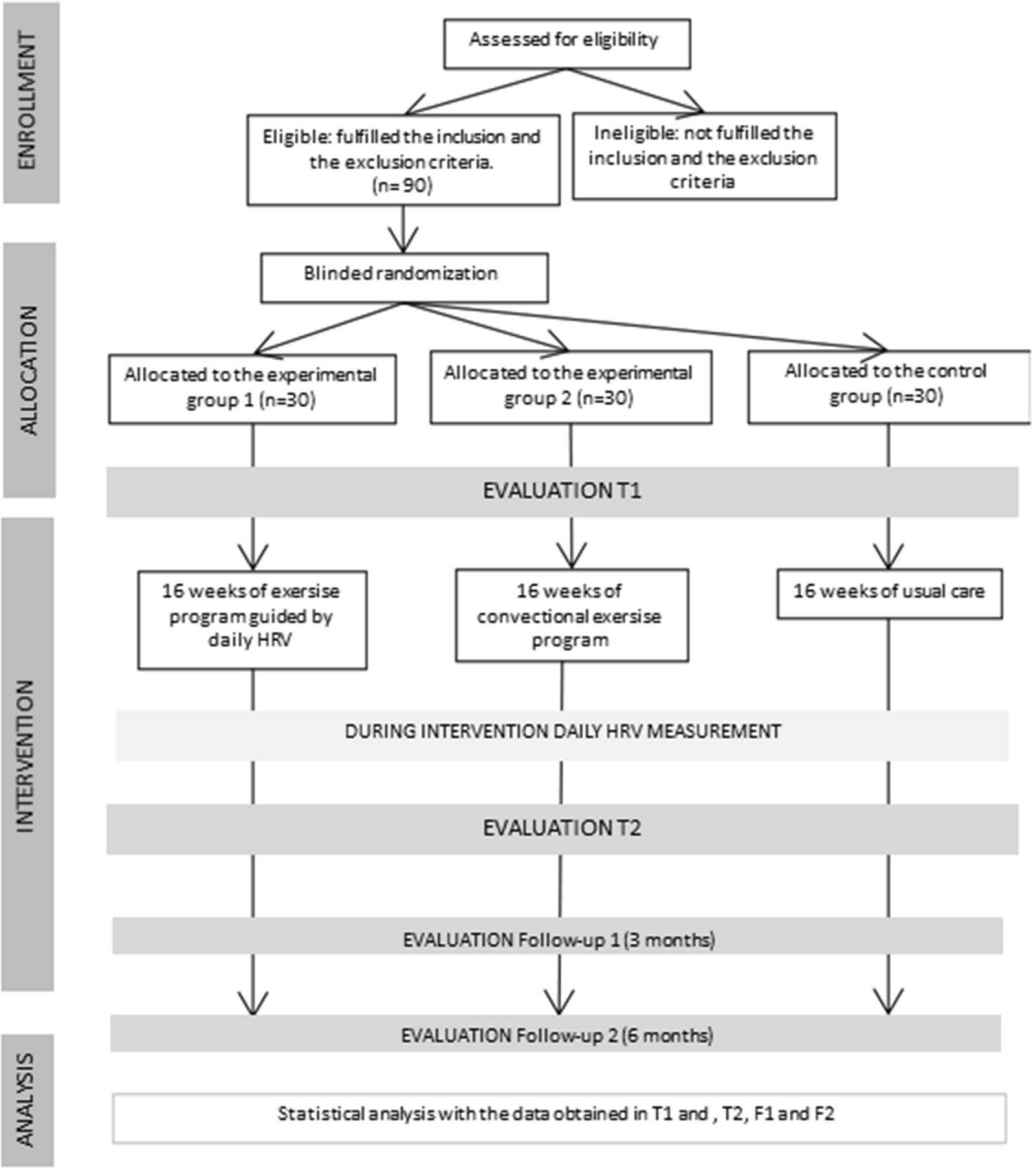
Study protocol timeline

The research will be conducted following the Helsinki declaration [43] and the American Society of Clinical Oncology policy statement [44]. Patients will be randomly assigned to three groups where two of them will participate in a physical exercise intervention and the left group will continue with usual care for 16 weeks (see Fig. 1). Moreover, the study methods described below follow the Standard Protocol Items Recommendations for Interventional Trials (SPIRIT) of 2013 [45] (SPIRT schedule and checklist are shown in the Additional file 1). After the positive response from the ethics committees, the trial was registered at ClinicalTrials.gov, recognized by the World Health Organization and the International Committee of Medical Journal Editors, under the identification number of NCT05040867.

### Participants

#### Recruitment and eligibility

A total of 90 patients are being recruited from the Oncology Service of Cantabria, concretely, they are being enrolled in the University Hospital of Marqués de Valdecilla. The enrolment consists of two phases. First, an oncologist and a radiologist select patients who fulfill the following inclusion criteria: (a) women aged between 18 and 65 years, (b) having luminal or triple-negative breast cancer, (c) having received and finished chemotherapy and radiotherapy treatment. In addition, patients are excluded if: (a) they have been scheduled for surgery during the study (b) they have HER2 + breast cancer, (c) they have severe heart disease before chemotherapy, (d) they have finished the cancer treatment more than 5 months ago, (e) they have metastatic cancer, (f) they have any injury which prevents them to perform the intervention or the evaluation correctly, (g) they have severe psychiatric illnesses.

In the second phase, patients are called to the hospital by the oncologist and the physical exercise professional to explain in detail the study and each intervention group requirement. After answering patients’ questions and asking for some technical needs for mobile phone application licenses, a complete description of the study is given to the participants.

#### Randomization and allocation

The study is a blinded, randomized, controlled trial. Before baseline assessments, participants are randomly assigned to one of the three groups: a group that performs the planned physical exercise program based on daily heart rate variability (HRVG), a group that performs the conventional pre-planned physical exercise program (PEG) and a control group (CG).

#### Ethical approval and registration

The design of this study complies with the Helsinki Declaration of 2014. Moreover, the ethical standards of the study have been approved by the ethical committee of the Rey Juan Carlos University (approval number: 1901202103121) and the ethical committee of the University Hospital of Marqués de Valdecilla, named Valdecilla Health Research Institute (IDIVAL) (register identification number: 42//2021). Both, the study registration and the positive ethics committee approval were carried out before the recruitment. Besides, all the participants are informed about the relevant aspects of this study before starting their program including the potential benefits and risks of practicing exercise. Any change in the protocol will be expressed on the clinical registration website of ClinicalTrials.gov.

#### Sample size

Given that, to our knowledge, no previous studies have examined the effects of an exercise program guided by daily HRV in breast cancer patients and its comparison to a moderate to high-intensity exercise group and a control group, the sample size was calculated by considering the exercise intensity differences between groups. In this regard, we have taken as reference values interventions carried out with three groups, a high-intensity group, a moderate-intensity group and a control group. Moreover, the oxygen consumption peak (VO_2peak_) has been selected to make the calculations due to its importance in cardiovascular and physical health. The chosen tool to analyze sample size has been using G-Power 3.1 and the calculation were done basing us on the data from the investigations carried out by Martin, Battaglini [46] and Northey, Pumpa [47] two different studies. In both cases, between-group significant differences were reported.

A total of 18 patients, (11 allocated to the experimental and 11 allocated to the control group) would be needed in the worst of the cases to achieve a 99% statistical power for VO_2peak_. Moreover, regarding the low number of participants obtain, the calculations concerning the effects of exercise in Global Longitudinal Strain (GLS) compared to a control group were also done. In this case, the data employed for the calculation was from Costello, Roberts [48] where significant differences within and between groups were achieved. So, in the prior calculations performed, we would need a total of 50 patients [25 in each group] to achieve a 99% statistical power (two tails). Therefore, assuming 20% of dropouts, 90 patients will be recruited and allocated to one group or the other. Due to the large number of patients that are estimable to be enrolled in the study, the recruitment and intervention will be carried out progressively over 24 months depending on the number of patients to be recruited.

### Interventions

#### Physical exercise programs

First, the common aspects between the two exercise groups are going to be mentioned, to explain afterwards the peculiarities of each physical exercise program. The programs of both experimental groups, PEG and HRVG, will last 14 weeks, and patients will train three times per week. All sessions will be supervised and remote. So that, each patient will connect with the physical exercise professional and with the other 4 participants by videocall. Moreover, the practitioner will monitor their heart rate in real-time because each patient will be given a MyZone heart rate (HR) control and platform access to MZ-Remote. In this way the professional will be able to adjust their workout intensity according to their HR, in the cardiovascular exercises and the weightlifting load in the resistance exercises; and to control and correct, participants’ posture and technique. Moreover, these measures will be determinant to asses participants’ exercise adherence. During the sessions, participants will be able to see their HR and HR of the rest of their training group. In addition, to promote social interaction an extra link will be given to those patients which want to see each other while practicing exercise. The necessary material to perform the exercise programs will be given to each participant in the before-intervention evaluation. This training kit will include a barbell of 2 kg, 2 weight discs of 5 kg, 2 weight discs of 2.5 kg, 2 weight discs of 1.25 kg and a Myzone's MZ-3 Belt chest strap.

Each session will last approximately 60 min and will include a warm-up, a main part composed of cardiovascular resistance exercises and a cooldown. The warm-up, lasting from 5 to 10 min, will include upper, lower body and truck mobility, and core muscular activation. The cooldown part will last about 5 min and will combine stretching of the muscles involved during the corresponding session. Moreover, during the stretches, breathing feedback will be given to fully relax and enhance elongation during the exhalation phase.

As for the main part of the sessions, lasting from 45 to 50 min approximately, the training schedule will be divided into three mesocycles. The first, with a duration of 4 weeks, will be focused on neuromuscular adaptation, learning exercise techniques and improving participants' aerobic capacity and HR self-control. In this regard, the exercises proposed in the period will be of low complexity and will be performed with the lowest program intensity. In the 5th week will begin the second mesocycle until week 10th when the intensity will increase progressively including exercises with greater technical complexity. In the last mesocycle from weeks 11th to 16th participants will experiment with the highest level of technical complexity and intensity archiving the highest weight load and cardiovascular intensity. In this way, the sessions, and their progression, will be designed according to the principles of training to achieve the greatest possible adaptations [49]. Concretely, during the first mesocycle, the participants will alternate two training circuits and during the second and third mesocycle patients will combine 3 different circuits, one each training day of the week. Part of the exercises has a cardiovascular component through intervallic training. These exercises will be combined with resistance training by performing them either at the beginning of the circuit, or between the strength exercises, or at the end of the strength circuit.

The cardiovascular component of the exercise program will be performed with different exercises such as: walking with knees up, jogging in place, skipping, sideways running by changing direction, jumping jacks, etc. The duration of the cardiovascular and its intervallic duration will vary in each mesocycle. In the first one, participants will perform 2 sets composed of 4 repetitions of 1 min with 45 s of rest between intervals and 2 min between sets. In the second and third mesocycles, the cardiovascular sets will be conducted between each resistance exercise. In this way, each circuit repetition will include 5 sets composed of 3 intervals of 30 s with 10 s of resting between repetitions in circuits 3, 4, 6 and 7. Whereas in circuits 5 and 8, which will be carried out on the third day of the training week of the second and third mesocycle, the intervals will be developed at the end of all the resistance exercises. Furthermore, every 6 weeks, on the third training day, the cardiovascular interval part will be done with dancing choreographies with the songs chosen by participants. Furthermore, every 5 weeks, on the third training day, the cardiovascular interval part will be done inside dancing choreographies with songs chosen by participants.

Regarding the resistance exercise of the training program, exercises will combine lower body strength with a predominance of both knees (e.g. squat or stride) and hip (e.g. deadlift or hip thrust) and upper body strength exercises of pushing (e.g. chest press, shoulder press or arm-triceps extension) and gripping (e.g. rowing or biceps curl). As mentioned, participants will progress in the complexity of the exercises, for example, from a squat starting from a chair to a barbell squat, or from a gluteal bridge to hip thrust. Table 1 shows a programming example that can serve as a reference for a better understanding of what the intervention will entail.

**Table 1:** Example of the main part of the physical exercise program

1st to 4 th week of exercise	
Circuit 1	Circuit 2
Walking rising knees (4 × 1 min) (2 sets) 1. Chair squat 2. Biceps curl 3. Lunge in place 4. Incline raw	Jog in place (4 × 1 min) (2 sets) 1. Hip trust 2. Triceps elbow extension 3. Chair squat 4. Chest press
Participants will repeat each circuit 4 times. Resistance exercise: 12 to 14 repetitions (HRVG) and 10 to 12 repetitions (PEG) in each circuit set. Rest: 20 s rest between exercises and 2 min between each of the laps of the circuit.	

5th to 10 th week of exercise			11th to 16th week of exercise		
Circuit 3	Circuit 4	Circuit 5	Circuit 6	Circuit 6	Circuit 7
1. Skipping 2. Biceps curl 3. Skipping 4. Triceps elbow extension 5. Skipping 6. Chest press 7. Skipping 8. Shoulder press	1. Jogging 2. Squat 3. Jogging 4. Dead lift 5. Jogging 6. Lunge 7. Jogging 8. Hip trust	1. Squat 2. Chest press 3. Hip trust 4. Incline raw 5. Lunge 6. Shoulder press Cardiovascular exercise*	1. Sideways running 2. Chest press 3. Sideways running 4. Shoulder press 5. Sideways 6. Biceps curl 7. Sideways running 8. Incline raw	1. Jumping jacks 2. Lunge 3. Jumping jacks 4. Dead lift 5. Jumping jacks 6. Squat 7. Jumping jacks 8. Hip trust	1. Squat 2. Shoulder press 3. Dead lift 4. Incline raw 5. Lunge 6. Chest press Cardiovascular exercise*
Participants will repeat each circuit 2 times Resistance exercises: 2 sets of 10–12 repetitions (HRVG) and 8–10 repetitions (PEG) Rest: 1 min rest between sets and 2 min between circuit repetition			Cardiovascular exercise: 3 sets os 30 s-interval + 10 s rest (circuits 3, 4, 6 and 7). 2 sets of 6 × 30 s-intervals repetitions + 10 s rest (circuits 5 and 8) * Dance activities once every 4 weeks		

##### Pre-planned remote moderate to high-intensity physical exercise program

The current exercise group will perform exercise following the recommendations of several and important literature such as the American College of Sports Medicine guidelines carrying the exercise with an intensity going from moderate to high intensity. Concretely in the cardiovascular exercise, the participant will start with an intensity of 65% of their reserve HR, in the first mesocycle; and will end with an intensity of 80% of their reserve HR. In the resistance exercise, they will commence lifting weights corresponding to their 55% RM and finish in the last mesocycle lifting loads matching their 70% RM.

##### High-intensity remote physical exercise program guided daily by HRV

Although all participants will daily measure their HRV, only in this intervention group each day's results will influence their exercise intensity. From the outcomes reported with smartphone photoplethysmography, explained the measurement process in the outcomes part, the root means squared differences of successive RR intervals (rMSSD) will be chosen as a reflection of vagal activity to prescribe the workout intensity [50]. In this regard, the natural logarithm of rMSSD will be calculated to make parametric statistical comparisons assuming a normal distribution. For establishing the training intensity and load, a 7-day rolling average measure (LrRMSSD7day-roll-avg) will be utilized. Subsequently, the scoring obtained will be contrasted in the smallest worthwhile change (SWC) to analyze if the daily result is inside SWC upper and lower limits, calculated as LnrMSSD reference week mean ± 0.5 × SD [51, 52]. The reference week of the first 4 training weeks will take the limits created in the baseline week, and in the rest of the program, an updated measure will be carried out every 4 weeks with the SWC for the past 4 weeks, because of the influence of exercise in participants cardiac autonomic modulation [53].

**Fig. 2:**
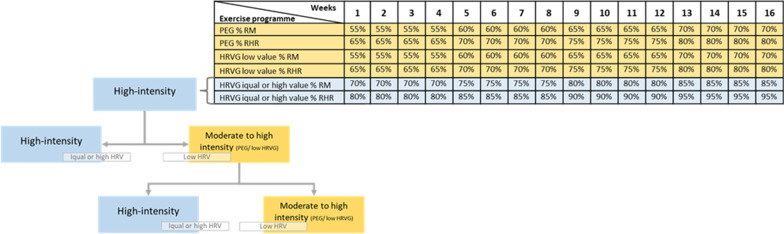
Intensity progression and intensity decision making process for the high-intensity exercise guide by HRV group

In this regard, when the daily LnrMSSD fell inside the limits created by the SWC patients will perform high-intensity training characterized by a cardiovascular intensity from 80 to 95% of their reserve HR (increasing a 5% every four weeks) and a weighting load from 70 to 85% of their RM (increasing the load 5% ever mesocycle, four weeks). Whereas if any day patients’ scoring fell out of the SWC, they will train at the same intensity as the PEG (Fig. 2).

#### Usual care control group

Participants who will be allocated to the CG will participate in the before and after intervention assessments will measure daily their HRV. They will be advised to continue with their regular activities of daily living without any exercise restrictions.

### Outcomes

The following outcomes will be assessed in person by the oncology group at the hospital (Marqués de Valdecilla Hospital) or by a physical exercise professional at the sports center (GOfit) when it corresponds. The evaluations will be carried out before, after the intervention and 3 and 6 months after the end of the intervention to all the participants (HRVG, PEG and CG). To ensure CG measurements and follow up evaluations the principal investigator will call or write participants each month.

#### Physiological outcomes

##### Cardiotoxicity measurements

Cardiotoxicity will be assessed at the hospital to have a more exhaustive control and safety of the participants and at the end of the program. On the one hand, ç hemograms will be carried out to assess of High-Sensitivity Cardiac Troponin, Troponin I, N-terminal portion of B-type natriuretic pro-peptide (NT-proBNP), Troponin T due to their predictive values of cardiac damage in patients under the effects of chemotherapy [54, 55]. Although these variables were primarily planned in the trial registration, the hospital will be only able to assess High-Sensitive Cardiac Troponin and NT-proBNP.

In addition, an echocardiogram will be performed to measure the left ventricular ejection fraction and GLS due to its modification caused by cardiotoxicity [56–58] together with mean and maximum aortic valve gradient, ventricles’ diameters and thicknesses and valve velocities because their possible aortic stenosis risk [59]. Moreover, a resting electrocardiogram will also be performed on each patient to obtain values for heart rate, heart rhythm, heart rate variability, I-axis and aVF, Q-T interval, QRS complex, S-T segment and T-wave [60].

##### Measurement of biomarkers

On the one hand, the measurement of the following biomarkers will be included: Tumor Necrosis Factor, interleukins IL-6, IL-8 and IL-1b, C-reactive protein, creatine kinase, global lactate dehydrogenase, alkaline phosphatase, bilirubin levels, monocyte chemotactic protein and vitamin D. On the other hand, the anti-inflammatory cytokines IL-1ra and IL-10 [61]. As occurred in the cardiotoxicity variables, the interleukins will not be able to be measured at the hospital.

##### Lipid profile and glucose measurement

Apart from the mentioned outcomes, other variables from the patients' blood tests related to cancer prognosis and cardiovascular risk will be taken. Triglycerides, low-density lipoproteins and high-density lipoproteins will be collected due to their association with cancer prognosis and mortality [62]. Relatedly, fasting glucose will be analyzed because of its relation to metabolic syndrome in breast cancer survivors [63]. Other serum and hematological analyses will be done to control exhaustively each of the patients.

##### Blood pressure and resting heart rate measurement

Blood pressure measurements will be taken using a validated oscillometer Omron Healthcare Oscillometer [64]. The participants will remain at rest for five minutes before the assessment and will be lying in a supine position with the legs and arms completed. They will place their left or right arm, depending on the affected breast, straight, so that the cuff is at the level of the heart, 2 cm from the elbow. In addition, any clothing that may alter the results will be removed. Once in this position, the air tube of the cuff will be placed on the front of the arm aligned with the middle finger and with the blue date on the cuff. Systolic and diastolic blood pressure data will be taken; and, subsequently, the mean arterial pressure will be calculated by the following formula [(systolic blood pressure + (2 × diastolic blood pressure))/3] [65].

The resting heart rate will be measured for 5 min. The participants will lie relaxed in the supine position on a stretcher isolated from the floor. This data will be essential for the correct prescription of the intensity.

##### Heart rate variability and heart rate in rest measurements

The assessment of heart rate in rest and HRV will be performed by the Polar H10 chest strap and by photoplethysmography (PPG) with the validated mobile app of HRV4training [50, 66]. The participants will remain in the supine position for 8 min to obtain 5 min of a stable signal. After removal of artifacts, by employing the Kubios® clinical software the temporal variables of standard deviation time domains of all RR intervals (SDNN), rMSSD, Average of all NN intervals (AVNN) and percentage of differences between adjacent NN intervals that are greater than 50 ms (pNN50) will be calculated [67]. Moreover, by also utilizing Kubios software the frequency measurements of low frequency (LF, 0.04–0.15 Hz), high frequency (HF, 0.15–0.4 Hz) and the LF/HF ratio will be obtained [67]. The HRV4Training app directly calculates the HRV outcomes.

On the other hand, daily heart rate variability (HRV) is measured using the HRV4Training application, a validated mobile application [50, 66] that allows HRV values to be obtained by PPG (rMSSD, LF, HF, SDNN, SDNN, AVNN, pNN50 heart rate and recovery points). The morning measurement will be performed every day, in the supine decubitus position upon awakening, for 1 min. During the basal week before starting the intervention, participants will measure their HRV, to establish the reference values for each participant of the GHRV group. In the intervention weeks, the participants will measure their HRV every morning, therefore, collecting morning HRV data (rMSSD, heart rate and recovery points). The value that will be taken into consideration to establish the GHRV training intensity will be the rMSSD as it represents the parasympathetic activation of the central nervous system. The detailed information of its utilization to exercise prescription is explained in the exercise program intervention part. To eliminate the placebo effect of the use of the application, the participants of the three physical exercise groups will perform this protocol. In this way, the HRV data of all the participants will be collected to analyze their evolution with the different interventions. To reduce measurement error, the application functioning is explained to all participants, mentioned to repeat the measurement if a message saying that the sign was not optimal or poor the need to repeat it. Moreover, the exercise professional will check the quality of the sign before its analysis.

##### Body composition measurement

The assessment of the percentage of fat and the visceral level of the participants are variables to be taken into consideration, due to the existing risk in women with overweight and obesity, of a greater relapse in breast cancer [68] and the possible effective role of high-intensity exercise [69]. Therefore, by utilizing the impedance Inbody analyzer [70] the body fat mass [68], body fat percentage, visceral fat level, musculoskeletal mass, body water, the bone mineral content, segmental fat mass (trunk, right and left arm and right and left leg) and segmental lean mass (trunk, right and left arm and right and left leg) of the patients will be evaluated [71]. In addition, all participants will be weighed and measured to obtain their Body Mass Index (BMI) by the formula of [weight/height^2^].

##### Measurement of anthropometric parameters

Patients will use a non-elastic tape measure for the evaluation of waist circumference, hip circumference, chest circumference, arm circumference, leg circumference following the guidelines set by the American College of Sports Medicine [72].

#### Physical exercise evaluations

##### Cardiorespiratory fitness measurement

To record the cardiorespiratory capacity, maximum heart rate and the patients' perception of effort, the Bruce incremental submaximal test (modified) [73] will be performed on a treadmill. During the test, the patients will wear a Polar H10 chest strap to monitor their heart rate. Thus, following the protocol created by Bruce, the treadmill will start with a 0% incline and a speed of 1.7 m/h which will be maintained during the first 3 min. Every 3 min both the speed and the incline will be increased until the participants decide that their fatigue is too high to continue. In addition to constant heart rate monitoring to record the maximum heart rate, the patient will be asked about her perception of exertion every minute. By acquiring the maximum heart rate we can then prescribe the target intensity from the reserve heart rate using Karvonen's formula [74]. At the end of the test, the recovery heart rate will be recorded 1 min after stopping the test in order to assess the recovery rate. Subsequently, the maximum oxygen consumption of each patient (VO_2max_ = 2.282* (time) + 8.545) will be calculated from the formula proposed by the creators. This submaximal test has been commonly used in cancer patients in different exercise interventions [75].

##### Lower and upper body strength measurement

Lower body strength resistance. For the measurement of the lower body strength resistance, the 30 s chair stand test will be used. The patients will use a chair without armrests, with the back against the wall so that it does not move during the test [76]. The evaluation will begin with the participant seated in the middle of the chair with arms flexed at the chest and hands resting on the opposite shoulder of the chair. The patient will stand up, positioning herself fully stretched, and will sit the maximum number of repetitions possible in 30 s. During the development, the patient will be encouraged and reminded of the need to sit down completely for each repetition. The number of repetitions achieved is recorded. This test has been performed previously in breast cancer survivors [37].

Upper body strength resistance. As for upper body strength- endurance, the patients will be measured by the 30-s arm curl test of the Senior fitness Manual [76]: a 2.5 kg weight will be used according to the Senior fitness Manual. The participants will be seated in a chair and with the dominant arm, they will have to perform elbow flexion extensions starting in full extension. The test will report the maximum number of repetitions in 30 s [76]. This assessment has been used previously in the breast cancer population [77, 78].

Lower and upper maximum force, velocity, and power. The evaluation of the maximum force will be carried out by using a linear encoder for the correct calculation of the maximum weight that the patient can lift in one repetition (RM) from the speed and power generated when moving the weight vertically. For the easy and correct performance, the different tests will be carried out on a multipower machine with a counterweight mechanism which will guide the vertical movement in a rectilinear way without the influence of the barbell weight.

The participants will begin to perform each of the exercises using the barbell as the only weight for 3 repetitions as a warm-up. After, at least three incremental weight discs will be added depending on the exercise and the patient's perception of effort and the speed of execution decline. At the end of each repetition, patients will be asked about their perception of perceived exertion using the modified Borg scale (Rate of Perceived Exertion, RPE) [79] and when this value has dropped to 2/10, the next weight will be added.

Diverse variables will be analyzed in each of the repetitions by employing the Chronojump by Boscosystem linear encoder, the MyLift mobile app [80] or the kinovea software [81] See supplementary table. So that, each participant’s repetition will be recorded by an iPhone camera of 240 frames per second.

This protocol will be repeated for each of the following exercises:

- Barbell Squat (Back Squat Test). Participants will hold the bar behind the back of their neck with the weight resting on their shoulders. If they have difficulty maintaining this position, the evaluation will be done by holding the bar in front of them under their neck with their arms crossed. Start with your knees fully extended and your feet shoulder-width apart. You will progressively bend your knees, without exceeding the line of the toes, until you reach 90° of flexion. Once you have reached this point, you will recover the initial position by extending your knees.
- Deadlift Test. Participants will begin the movement with feet hip-width apart, hands in the centre of the bar (placed on the floor) and shoulder-width apart, the bar will be placed close to the shins and the knees bent until the hands can grasp the bar. The chest should remain upright and the back straight throughout the exercise. The hips should be above the knee so that the shoulders are positioned in front of the bar. The movement starts with a slight knee extension, followed by a hip extension and ends with a simultaneous knee and hip extension until the shoulders are positioned behind the bar, thus aligning the whole body [82].
- Lunge Test. Participants will start the movement by placing their feet shoulder-width apart, holding the bar behind the back of their neck, leaving the weight of the bar on their shoulders. They will take one step forward. The distance of this will correspond to the distance from the superior iliac spine to the medial malleolus of the tibia. Once this distance is reached, bend the knees, touching the floor with the back knee and reaching 90° of flexion in the front knee. Subsequently, they will recover the initial position by fully extending the front leg and progressively transferring the weight of the body to the back leg [83].
- Chest press (Chess press). Participants lie on the bench with their legs bent to the sides of the bench at slightly more than 90°. They will grasp the bar with their hands wider than shoulder-width apart. The movement will begin with the elbows fully extended and with the bar at throat level. Progressively lower the bar by bending the shoulders, followed by bending the elbows until the bar reaches one centimeter from the chest. Once the maximum flexion is achieved, they will extend the elbows and shoulders until they return to the initial position [37, 84].
- Shoulder press. Participants sit on the bench with their backs against the backrest. Start the movement by grasping the bar with elbows fully extended with hands 5 cm wider than shoulder-width apart. Lower the bar continuously until it comes into contact with the upper chest. Then start the concentric phase by extending the elbows completely to the starting position [85].
- Bent over row. Participants perform the exercise with their backs bent at 45°. They begin the movement by holding the bar in front of their shoulders with their hands shoulder-width apart. Proceed to bend the elbows, bringing them behind the body until the barbell touches the middle of the abdomen [86].
- Biceps curl with barbell. Participants will stand in an anatomical position with knees slightly bent and feet apart. They will hold the bar with a supine grip, arms parallel to the sides of the body. Begin the movement with elbows fully extended and progressively bend the elbows to their maximum. Subsequently, they will extend their elbows to the starting position [87].

Finally, the strength-power will be measured by employing a countermovement jump test (CMJ) and from squat (SJ) to evaluate the explosiveness of the patient, as has been done in previous studies on patients with breast cancer [88]. it was decided to eliminate the assessment of hip trust and triceps barbell strength, included in the clinical registration because performing all the tests would take too long for the patients.

Vertical applied force. The force-power will be measured utilizing a CMJ and from SJ as a reflection of the application of force in the unit of time, inter and intramuscular coordination and the use of the elastic energy of those actions in which the stretch–shortening cycle of the lower limbs predominates, which is present in the majority of actions in daily life, as has been carried out in previous studies in patients with breast cancer [88]. In the evaluations after the intervention, those patients in whom it is possible to evaluate the jump with progressive loads, the vertical force–velocity profile will be performed, which will give us information on how the subject is currently producing power and how it should (based on their characteristics) in order to be able to evaluate the explosiveness of the patients [89]. To obtain it, together with the SJ and CMJ jumps without load, the patients will perform 2–3 more attempts with progressive loads, determining the increase in load according to the height of the jump without weight [90]. In addition, for the corresponding calculations, two body measurements will be taken to determine the push-off distance (pod). For the evaluation of the different variables indicated, different technologies will be used such as the Chronojump’s contact platform and the recording with an IOS camera of 240 f.p.s of each of the jumps for subsequent analysis with the validated application of My Jump [91] and Kinovea [92].

 Horizontal applied force. For the evaluation of this manifestation, of force, an acceleration action will be recorded (the only requirement is to start from speed 0 and to cover a distance of 30 m in the shortest possible time, the time used not being a limitation for the performance of the test). This recording will be used to calculate the horizontal force–velocity, which will give a mechanical description of how the patient has applied forces to move horizontally. For the complete assessment of the horizontal profile in the post-intervention evaluation, patients will perform 3 progressive 30 m sprints by running or walking (at 50%, 70% and 90% of the participants' maximum self-perceived speed) [93]. For this, an IOS device with 240 frames per second will be used and subsequently analysed with the validated App for IOS devices (My Sprint) [94]. The device, iPhone will be mounted to a tripod (in the frontal plane) to film the sprint from the side, at the 15 m marker and at 18 m from the track, to register the entire sprint. Since the iPhone 6 was in a fixed position, video parallax was corrected to ensure 5 m, 10 m-, 15 m-, 20 m-, 25 m and 30 m split times were measured properly.

##### Measurement of agility and balance

The Timed up and go test will be employed to assess patients’ agility as it has been done before in breast cancer patients [95]. For its performance, a chair will be placed against the wall and a cone will be placed in 3 m from the front edge of the chair to the back of the cone. The patient will sit in the middle of the chair keeping her back straight, her hands on her thighs and both feet on the floor. At the "go" signal, the participants will get up from the chair and walk as fast as possible to the cone, and around the cone, back to the chair. The time taken to complete the test will be recorded. The test will be repeated twice, and the best score achieved.

The One-leg stand test belonging to the ALPHA battery in adults [96] will be used to evaluate static balance. To do this, the patient will be placed in monopodal support, placing the sole on the inside of the knee. The test will be performed first with the right feet on the ground and then with the left. Patients will be able to use their arms to balance only when necessary.

##### Lower and upper body flexibility measurement

Hamstring flexibility will be measured using the V Sit and Reach test [8] where the participant will be seated on the floor with knees fully extended and feet 30 cm apart. Place a tape measure on the floor, equidistant between the feet, placing the 0 cm in line with the heels. Putting the palms of the hands together and keeping the elbows fully extended, the participants will bend their trunks as much as possible on the tape measure. The missing centimetres or the centimeters that are passed from their maximum bending point and the 0 cm will be noted.

Upper body flexibility will be evaluated by utilizing the Back scratch test, previously used in breast cancer patients [95] and belonging to the Senior Fitness Test battery [76]. During the test, the participants will be placed in a standing position, having to try to reach above their head, with their arm in flexion and external rotation, to touch their other hand, which will be placed on their back in supine position with the arm flexed [95]. The test will be performed first with the arm through which the chemotherapy was administered and then repeated with the other arm.).

#### Psychosocial and physical exercise outcomes

Psychological variables will be assessed utilizing self-administered questionnaires specifically validated for cancer patients.

##### Health-related quality of life measurement

To assess HRQoL patients will complete the European Organisation for Research and Treatment of Cancer Quality of Life Questionnaire (EORTC QLQ-C30) [97] which includes functional and cancer-specific symptom assessment and the results will be analyzed following the instructions given in its manual [98]. Concretely, the QLQ-C30 is composed of both multi-item scales to assess functional, symptoms and global health status and six single-item measures to score the left symptoms. Inside the functional outcomes, 6 items correspond to physical function, 2 to functional role, 4 to emotional function, 2 to cognitive function and 2 to a social function. Moreover, the assessed symptoms will be fatigue (3 items), nausea and vomiting (2 items), pain (2 items), dyspnoea (1 item) insomnia (1 item), appetite loss (1 item), constipation (1 item), diarrhea (1 item) and financial difficulties (1 item). All the scales and single-item measures range in score from 0 to 100. A high scale score represents a higher response level. Thus, a high score for a functional scale represents a high/healthy level of functioning, a high score for the global health status / QoL represents a high QoL, but a high score for a symptom scale/item represents a high level of symptomatology/problems [23].

##### Cancer-related fatigue measurement

For the assessment of cancer-related fatigue, the fatigue subscale of Functional Assessment of Chronic Illness Therapy- Fatigue questionnaire [99] will also be used and analyzed according to the instructions of its creators [100]. The questionnaire is composed of 13 items where participants would answer a Likert scale of 4 points being 1 “Not at all” and 4 “Very much”. The questions are asked about perceived fatigue in the last 7 days. The score could range from 0 to 52, so that, higher scorings represent high fatigue symptoms.

##### Life satisfaction, self-esteem, depression and anxiety measurements

Life satisfaction will be assessed using the Satisfaction With Life Scale questionnaire [101], validated in Spanish breast cancer patients [102]. The scale is composed of 5 items to answer with a Likert scale from 1 to 5 being 1 “strongly disagree” and 5 “strongly agree”. Higher scores mean higher satisfaction with life [101]. In addition, the Rosenberg Self-Esteem Scale [103] employed before in cancer patients [104] will be utilized for analyzing Spanish breast cancer patients’ self-esteem. The scale, validated in Spanish [105], comprises ten items with a Likert scale of four options. According to the sum of their items, the scale is divided into three ranges: normal or high self-esteem (between 30 and 40 points), medium self-esteem (26–29 points), and low self-esteem (< 26 points). It has a Cronbach’s alpha of 0.87 and a reliability of 0.74 [105].

In addition, the Hospital Anxiety and Depression (HAD) Scale will be used and analyzed according to the authors' indications [106]. This questionnaire was validated in Spanish oncological patients to obtain results regarding anxiety and depression [107]. The HAD scale consists of 14 items referring to participants’ perceptions regarding the last week. The odd-numbered items make up the anxiety subscale and their response scale are scored from 3 to 0. The even-numbered items make up the subscale of depression and are scored from 0 to 3. The total score in each subscale is obtained by adding the scores of the corresponding items, with a range each from 0 to 21. In both cases, the higher the score, the higher the level of anxiety or depression [106].

##### Shoulder mobility disability and kinesiophobia measurements

For the assessment of their perception of disability due to shoulder mobility so common after breast cancer treatment, the Quick Disabilities of the Arm, Shoulder and Hand questionnaire will be used [108, 109]. The scale is composed of 11 items graded from 0 to 100 asking patients to rate the level of difficulty and pain in performing several tasks over the past week. Patients will answer each question scoring from 1 to 5 selecting 1 when the activities are performed with no difficulty and 5 for those activities unable to perform with “extreme difficulty”. Relatedly, fear of movement will be measured using the 11-items Tampa Kinesiophobia Scale [110, 111]. The scale has two subscales, one focused on activity avoidances (reflecting the belief that activity may result in injury or increased pain) and the other concerning somatic focus (indicating the belief in underlying and serious medical problems). The total score ranges from 11–44 points with higher scores indicating greater fear of pain, movement, and injury [111]. Items on the TSK-11 are scored from 1 (strongly disagree) to 4 (strongly agree) [111].

##### Physical activity and motivation for exercising measurements

Finally, the International Physical Activity Questionnaire be administered to evaluate the level of physical activity of the patients in the assessment periods [112]. The 8 questions refer to the time patients spent being active in the last 7 days to analyze the time spent in making low-intensity activities, moderate-intensity activities, high-intensity activities and sedentary activities [112]. In addition, the existence or not of behavioral change/motivation towards exercise will be assessed using the Behavioural Regulation of Exercise Behaviour Scale—2 [113]. This questionnaire is composed of a 19-item scale with five factors (amotivation, external, introjected, identified and intrinsic motivation) [114]. Patients will reflect their opinion by a Likert scale from 1 to 5 corresponding 1 to “strongly disagree” and 5 to “strongly agree”.

### Statistical analysis

All the outcome results will be included in an anonymous database to conduct the statistical analyses. A descriptive, quantitative, and graphics analysis will be performed of all the included variables. The statistic software employed will be the IBM Statistical Package for the Social Sciences (version 25.0; SPSS, Inc., Chicago, IL, USA) [115]. The number of statistical analyses will be high and diverse depending on the nature of the results. In general, we will proceed with the usual steps to check the normality and homogeneity of variables.

First, the Kolmogorov–Smirnov and Shapiro Wilk tests will be used to determine whether parametric or non-parametric statistical analyses should be applied to each of the variables analyzed. In this regard, normal distribution will be assumed when the p-value is greater than 0.05 indicating that the variable tested was not significantly different from a normal distribution. Descriptive results will be presented as mean and SD or median (range) according to the results of the normality tests. Subsequently, to evaluate the effects of the program, a repeated-measures ANOVA or Kruskal–Wallis, depending on the normal distribution, will be used. The interaction factors of the ANOVA, or Kruskal–Wallis, will be the group (HRVG, PEG or CG) and the timing of the measure (before, after the intervention and follow-up). Besides, a Bonferroni analysis will be performed to adjust the results. Results shall include the Cohen’s d effect size (95% confidence interval) and statistical significance for each dependent variable concerning time and its interaction effects (group × time). Moreover, to analyze the differences between the group independently in each of the variables, the T-test for Independent Samples and the T-test for related samples, or a Mann–Whitney U-test or Wilcoxon in case of not fulfilling the principle of normality, will be employed.

Concurrently, a correlation analysis is planned to be done to establish novel relations between some of the measures performed. For instance, a Pearson o Spearman analysis (depending on the results of the normal distribution analysis) will be calculated between the change in strength, the body composition, cardiotoxicity variables and the psychological variables. Moreover, a correlation between the results obtained in the different questionnaires will be calculated.

## Discussion

The present study will be the first, as far as we are concerned, on evaluating the physiological, physical and psychosocial effects of high-intensity exercise guided by HRV in contrast to pre-planned exercise and usual care. In addition, it will also distinguish because of its novelty in analyzing cardiotoxicity effects, together with a detailed physiological, physical and psychosocial study, in high-intensity exercise versus moderate to high intensity and in performing a remote, but supervised in real-time, intervention.

First, as has been mentioned in the article, all the patients will register daily their HRV measurements. Some studies have been carried out before analyzing the effects of exercise in autonomic modulation variables in breast cancer patients but none of them have registered daily participants' measurements [116]. In a meta-analysis recently published, the data obtained showed significant differences between the exercise and the control groups in all the HRV variables analyzed (SDNN, rMSSD, LF, HF, LF/HF ratio) although it could be noticed that interventions involving higher intensities reached greater effects [116]. In this regard, as in the current study patients in the HRVG will perform high-intensity training, we expect to get at least the same HRV positive modifications or better due to the higher intensity reached by the meta-analysis participants was 60% VO_2max_ [117, 118] or 13–14 RPE [119, 120].

Moreover, as it has been developed before in athletes [39], but not in clinical populations, the intensity of HRVG participants will be adjusted according to rMSSD values, as a reflection of the parasympathetic activity. rMSSD suffers normally a decrease during and after cancer treatments [121, 122], which exercise may regain by a decrease of renin and angiotensin, affecting the renin–angiotensin–aldosterone system; a decline of oxidative stress and inflammation caused by the increase in catecholamines impacting, therefore, in tumour cell microenvironment by an exponential decrease of reactive oxidative species and an increment in antioxidants [61, 123–126]. In this way, to get an rMSSD increase and adapt to their training load to their physiological stress, if patients' rMSSD goes beyond their individual limits, they will not perform in those days high-intensity and will follow PEG intensity until their values return to their corresponding normality [127]. This novelty way to prescribe and adapt high-intensity exercise to patients daily could provide a new tool to reach higher benefits. Two recent meta-analyses have stated that high-intensity exercise does not produce higher positive changes in the VO_2max_ [23] and HRQoL [26] of cancer patients than moderate intensity. In this regard, we hypothesize that maybe not all patients reacted equally to high-intensity, and they need more individualization concerning their disease's long-term side effects, especially the related cardiovascular abnormalities.

Relatedly, the second aspect to highlight is the novelty of the exercise interventions compared to ones perform so far analyzing cardiotoxicity variables. To date, from our perspective, is the first study that will apply high and moderate to high-intensity cardiovascular and resistance training in breast cancer patients to analyze the cardiotoxicity effects, among others. Furthermore, except for one study protocol recently published [128] and a few studies in rats [129, 130], it would be also one of the first of including resistance training with so high weight and volume loads. Moreover, regarding exercise timing, the exercise programs focused on the improvement of cardiotoxicity variables such as LVEF, GLS, or troponin, most of the studies are carried out during breast cancer patients’ treatments [131] and not after them as our study will perform. However, cardiac abnormalities in breast cancer patients may appear within the first year after the end of chemotherapy, in case of early cardiotoxicity, or after several years following the completion of treatment (median of 7 years) [4]. Accordingly, to improve patients' cardiorespiratory fitness, troponin and GLS values and maintain the levels reached by acquiring physical exercise habits, in the current study patients have recently finished chemotherapy and radiotherapy to prevent the potential early and late cardiotoxicity. We will try to teach patients how to train during the 16 weeks of the exercise programs and to generate positive and self-determination experiences through exercise [132]. So that, we will promote exercise adherence to continue exercising even after its end knowing their cardiovascular and resistance levels at the end of the programs. Moreover, maybe if exercise programs are developed uniquely during chemotherapy or before it, patients can associate exercise to unpleasant cancer treatments or phases being unlikely to maintain exercising after treatments.

The final aspect of the study to be highlighted, but not less important, is the novelty of the exercise programs modalities. Common exercise interventions are carried out supervised by in-person training or unsupervised by home-based exercise, but none have involved remotely supervised exercise programs. The COVID-19 situation has forced physical professionals to adapt exercise programs to online supervision, whereas in breast cancer patients, only a case study has reported its development during the pandemic where, by video calls, two patients made cardiovascular and resistance training with the intensity controlled by HR monitors controlled by patients [133]. In this line, another intervention, involving aerobic exercises, was carried out in-person at the begging, but the covid situation restrictions obliged them to transform the rest of the intervention supervised remotely by video calls [134]. In contrast, our exercise interventions are supervised from the beginning and HR is monitored in real-time by the physical professional. However, the rest of the home-based investigations, to the best of our knowledge, are not supervised by video calls, so real-time corrections and HR control are not commonly done. In these cases, interventions are usually based on physical activity recommendations or aerobic exercise such as walking [135] and very few include resistance training [136, 137] where counseling is performed by weekly calls or diaries, exercise is controlled by RPE or pedometer [137] and exercises intensity is commonly prescribed from low to moderate intensity. In this regard, the only home-based high-intensity intervention, carried out unsupervised-unlike ours-, was developed by Ochi, Tsuji [138] where patients trained for 10 min 3 times per week including bodyweight exercises in a smartphone application.

## Supplementary Information

**Additional file 1. Table S1**. SPIRIT schedule of study’s enrolment, interventions, and assessments.** Table S2**. SPIRIT check list for the study.** Table S3**. Overview of all the study variables with the corresponding measurement tool.

## Data Availability

All the needed additional material, without breaching participant confidentiality, will be freely available for non-commercial purposes. The datasets used and/or analysed during the current study are available from the corresponding author on reasonable request.

## Abbreviations

HRV: Heart rate variability
HRQoL: Health related quality of life
SPIRIT: Standard Protocol Items Recommendations for Interventional Trials
BNP: Brain natriuretic peptide
RM: 1-Repetion Maximum
HER2: Human epidermal growth factor receptor 2
HRVG: Physical exercise programme group guide by heart rate variability
PEG: Pre-planned physical exercise programme group
CG: Control group
IDIVAL: Instituto de investigación sanitaria Valdecilla
VO_2peak_: Oxygen consumption peak
GLS: Global longitudinal strain
LVEF: Left ventricular ejection fraction
HR: Heart rate
rMSSD: Root means squared differences of successive RR intervals
SWC: Smallest worthwhile change
NT-proBNP: N-terminal portion of B-type natriuretic pro-peptide
SDNN: Standard deviation time domains of all RR intervals
AVNN: Average of all NN intervals
pNN50: Percentage of differences between adjacent NN intervals that are greater than 50 ms
RPE: Rate of perceived exertion
CMJ: Countermovement jump
SJ: Squat jump
EORTC QLQ-C30: European Organisation for Research and Treatment of Cancer Quality of Life Questionnaire
HAD: Hospital anxiety and depression

## References

[1] Bluethmann SM, Mariotto AB, Rowland JH (2016). Anticipating the "Silver Tsunami": prevalence trajectories and comorbidity burden among older cancer survivors in the United States. Cancer Epidemiol Biomark Prev Publ Am Assoc Cancer Res Cosponsored Am Soc Prev Oncol.

[2] Ramin C, Schaeffer ML, Zheng Z, Connor AE, Hoffman-Bolton J, Lau B (2020). All-cause and cardiovascular disease mortality among breast cancer survivors in CLUE II, a long-standing community-based cohort. JNCI J Natl Cancer Inst.

[3] Shapiro CL (2018). Cancer survivorship. N Engl J Med.

[4] Saleh Y, Abdelkarim O, Herzallah K, Abela GS (2021). Anthracycline-induced cardiotoxicity: mechanisms of action, incidence, risk factors, prevention, and treatment. Heart Fail Rev.

[5] Coumbe BG, Groarke JD (2018). Cardiovascular autonomic dysfunction in patients with cancer. Curr Cardiol Rep.

[6] Prado CM, Baracos VE, McCargar LJ, Reiman T, Mourtzakis M, Tonkin K (2009). Sarcopenia as a determinant of chemotherapy toxicity and time to tumor progression in metastatic breast cancer patients receiving capecitabine treatment. Clin Cancer Res.

[7] Shachar SS, Deal AM, Weinberg M, Nyrop KA, Williams GR, Nishijima TF (2017). Skeletal muscle measures as predictors of toxicity, hospitalization, and survival in patients with metastatic breast cancer receiving taxane-based chemotherapy. Clin Cancer Res.

[8] Lovelace DL, McDaniel LR, Golden D (2019). Long-term effects of breast cancer surgery, treatment, and survivor care. J Midwifery Womens Health.

[9] Foulkes SJ, Howden EJ, Bigaran A, Janssens K, Antill Y, Loi S (2019). Persistent Impairment in cardiopulmonary fitness after breast cancer chemotherapy. Med Sci Sports Exerc.

[10] Jensen MT, Holtermann A, Bay H, Gyntelberg F (2017). Cardiorespiratory fitness and death from cancer: a 42-year follow-up from the Copenhagen Male Study. Br J Sports Med.

[11] Zhang X-M, Dou Q-L, Zeng Y, Yang Y, Cheng AS, Zhang W-W (2020). Sarcopenia as a predictor of mortality in women with breast cancer: a meta-analysis and systematic review. BMC Cancer.

[12] De Couck M, Mravec B, Gidron Y (2012). You may need the vagus nerve to understand pathophysiology and to treat diseases. Clin Sci.

[13] Robsahm TE, Falk RS, Heir T, Sandvik L, Vos L, Erikssen JE (2016). Measured cardiorespiratory fitness and self-reported physical activity: associations with cancer risk and death in a long-term prospective cohort study. Cancer Med.

[14] Antoun S, Borget I, Lanoy E (2013). Impact of sarcopenia on the prognosis and treatment toxicities in patients diagnosed with cancer. Curr Opin Support Palliat Care.

[15] West MA, Asher R, Browning M, Minto G, Swart M, Richardson K (2016). Validation of preoperative cardiopulmonary exercise testing-derived variables to predict in-hospital morbidity after major colorectal surgery. Br J Surg.

[16] Frye JN, Sutterfield SL, Caldwell JT (2018). Vascular and autonomic changes in adult cancer patients receiving anticancer chemotherapy. J Appl Physiol.

[17] Lakoski SG, Jones LW, Krone RJ, Stein PK, Scott JM (2015). Autonomic dysfunction in early breast cancer: incidence, clinical importance, and underlying mechanisms. Am Heart J.

[18] Strongman H, Gadd S, Matthews A, Mansfield KE, Stanway S, Lyon AR (2019). Medium and long-term risks of specific cardiovascular diseases in survivors of 20 adult cancers: a population-based cohort study using multiple linked UK electronic health records databases. The Lancet.

[19] Abdel-Qadir H, Thavendiranathan P, Austin PC, Lee DS, Amir E, Tu JV (2019). The risk of heart failure and other cardiovascular hospitalizations after early stage breast cancer: a matched cohort study. JNCI J Natl Cancer Inst..

[20] Coumbe BG, Groarke JD (2018). Cardiovascular autonomic dysfunction in patients with cancer. Curr Cardiol Rep.

[21] Ramin C, Schaeffer ML, Zheng Z, Connor AE, Hoffman-Bolton J, Lau B (2021). All-cause and cardiovascular disease mortality among breast cancer survivors in CLUE II, a long-standing community-based cohort. JNCI J Natl Cancer Inst..

[22] Ginzac A, Passildas J, Gadéa E, Abrial C, Molnar I, Trésorier R (2019). Treatment-induced cardiotoxicity in breast cancer: a review of the interest of practicing a physical activity. Oncology.

[23] Lavín-Pérez AM, Collado-Mateo D, Mayo X, Humphreys L, Liguori G, James Copeland R (2021). High-intensity exercise to improve cardiorespiratory fitness in cancer patients and survivors: a systematic review and meta-analysis. Scand J Med Sci Sports.

[24] Antunes P, Esteves D, Nunes C, Amarelo A, Fonseca-Moutinho J, Afreixo V (2021). Effects of exercise on cardiac function outcomes in women receiving anthracycline or Trastuzumab treatment for breast cancer: a systematic review and meta-analysis. Appl Sci.

[25] Varghese SS, Johnston WJ, Eekhoudt CR, Keats MR, Jassal DS, Grandy SA (2021). Exercise to reduce anthracycline-mediated cardiovascular complications in breast cancer survivors. Curr Oncol.

[26] Lavín-Pérez A, Collado-Mateo D, Mayo X, Liguori G, Humphreys L, Copeland R (2021). Effects of high-intensity training on the quality of life of cancer patients and survivors: a systematic review with meta-analysis. Sci Rep.

[27] Feeney LR, Tormey SM, Harmon DC (2018). Breast cancer and chronic pain: a mixed methods review. Ir J Med Sci.

[28] van Hall G (2010). Lactate kinetics in human tissues at rest and during exercise. Acta Physiol (Oxf).

[29] San-Millán I, Brooks GA (2016). Reexamining cancer metabolism: lactate production for carcinogenesis could be the purpose and explanation of the Warburg Effect. Carcinogenesis.

[30] Papadopoulos E, Santa Mina D (2018). Can we HIIT cancer if we attack inflammation?. Cancer Causes Control.

[31] Villaseñor A, Ballard-Barbash R, Baumgartner K, Baumgartner R, Bernstein L, McTiernan A (2012). Prevalence and prognostic effect of sarcopenia in breast cancer survivors: the HEAL Study. J Cancer Surv Research Pract.

[32] Au PC-M, Li H-L, Lee GK-Y, Li GH-Y, Chan M, Cheung BM-Y (2021). Sarcopenia and mortality in cancer: a meta-analysis. Osteoporos Sarcopenia..

[33] Freedman RJ, Aziz N, Albanes D, Hartman T, Danforth D, Hill S (2004). Weight and body composition changes during and after adjuvant chemotherapy in women with breast cancer. J Clin Endocrinol Metab.

[34] Prado CM, Baracos VE, McCargar LJ, Reiman T, Mourtzakis M, Tonkin K (2009). Sarcopenia as a determinant of chemotherapy toxicity and time to tumor progression in metastatic breast cancer patients receiving capecitabine treatment. Clin Cancer Res Off J Am Assoc Cancer Res.

[35] Malietzis G, Currie AC, Athanasiou T, Johns N, Anyamene N, Glynne-Jones R (2016). Influence of body composition profile on outcomes following colorectal cancer surgery. Br J Surg.

[36] Clifford B, Koizumi S, Wewege MA, Leake HB, Ha L, Macdonald E (2021). The effect of resistance training on body composition during and after cancer treatment: a systematic review and meta-analysis. Sports Med.

[37] Montaño-Rojas LS, Romero-Pérez EM, Medina-Pérez C, Reguera-García MM (2020). Resistance training in breast cancer survivors: a systematic review of exercise programs. Int J Environ Res Public Health.

[38] Jones LM, Stoner L, Baldi JC, McLaren B (2020). Circuit resistance training and cardiovascular health in breast cancer survivors. Eur J Cancer Care.

[39] Javaloyes A, Sarabia JM, Lamberts RP, Plews D, Moya-Ramon M (2020). Training prescription guided by heart rate variability vs. block periodization in well-trained cyclists. J Strength Cond Res.

[40] Guo Y, Palmer JL, Strasser F, Yusuf SW, Bruera E (2013). Heart rate variability as a measure of autonomic dysfunction in men with advanced cancer. Eur J Cancer Care.

[41] Forte G, Troisi G, Pazzaglia M, Pascalis VD, Casagrande M (2022). Heart rate variability and pain: a systematic review. Brain Sci.

[42] Sotirova MB, McCaughan EM, Ramsey L, Flannagan C, Kerr DP, O'Connor SR (2021). Acceptability of online exercise-based interventions after breast cancer surgery: systematic review and narrative synthesis. J Cancer Surv Res Pract.

[43] World Medical Association Declaration of Helsinki (2014). World Medical Association Declaration of Helsinki ethical principles for medical research involving human subjects. J Am Coll Dent.

[44] American Society of Clinical Oncology (2003). American Society of Clinical Oncology policy statement: oversight of clinical research. J Clin Oncol Off J Am Soc Clin Oncol.

[45] Chan A-W, Tetzlaff JM, Altman DG, Laupacis A, Gøtzsche PC, Krleža-Jerić K (2013). SPIRIT 2013 statement: defining standard protocol items for clinical trials. Ann Intern Med.

[46] Martin EA, Battaglini CL, Hands B, Naumann F (2015). Higher-intensity exercise results in more sustainable improvements for VO2peak for breast and prostate cancer survivors. Oncol Nurs Forum.

[47] Northey JM, Pumpa KL, Quinlan C, Ikin A, Toohey K, Smee DJ (2019). Cognition in breast cancer survivors: a pilot study of interval and continuous exercise. J Sci Med Sport.

[48] Costello BT, Roberts TJ, Howden EJ, Bigaran A, Foulkes SJ, Beaudry RI (2019). Exercise attenuates cardiotoxicity of anthracycline chemotherapy measured by global longitudinal strain. Cardiol Oncol.

[49] Campbell KL, Neil SE, Winters-Stone KM (2012). Review of exercise studies in breast cancer survivors: attention to principles of exercise training. Br J Sports Med.

[50] Plews DJ, Scott B, Altini M, Wood M, Kilding AE, Laursen PB (2017). Comparison of heart-rate-variability recording with smartphone photoplethysmography, Polar H7 chest strap, and electrocardiography. Int J Sports Physiol Perform.

[51] Kiviniemi AM, Hautala AJ, Kinnunen H, Nissilä J, Virtanen P, Karjalainen J (2010). Daily exercise prescription on the basis of HR variability among men and women. Med Sci Sports Exerc.

[52] Plews DJ, Laursen PB, Kilding AE, Buchheit M (2012). Heart rate variability in elite triathletes, is variation in variability the key to effective training? A case comparison. Eur J Appl Physiol.

[53] Bellenger CR, Fuller JT, Thomson RL, Davison K, Robertson EY, Buckley JD (2016). Monitoring athletic training status through autonomic heart rate regulation: a systematic review and meta-analysis. Sports Med.

[54] Sparano JA, Wolff AC, Brown D (2000). Troponins for predicting cardiotoxicity from cancer therapy. Lancet.

[55] Morris PG, Chen C, Steingart R, Fleisher M, Lin N, Moy B (2011). Troponin I and C-reactive protein are commonly detected in patients with breast cancer treated with dose-dense chemotherapy incorporating trastuzumab and lapatinib. Clin Cancer Res.

[56] Demissei BG, Hubbard RA, Zhang L, Smith AM, Sheline K, McDonald C (2020). Changes in cardiovascular biomarkers with breast cancer therapy and associations with cardiac dysfunction. J Am Heart Assoc.

[57] Oikonomou EK, Kokkinidis DG, Kampaktsis PN, Amir EA, Marwick TH, Gupta D (2019). Assessment of prognostic value of left ventricular global longitudinal strain for early prediction of chemotherapy-induced cardiotoxicity: a systematic review and meta-analysis. JAMA Cardiol.

[58] Howden EJ, Bigaran A, Beaudry R, Fraser S, Selig S, Foulkes S (2019). Exercise as a diagnostic and therapeutic tool for the prevention of cardiovascular dysfunction in breast cancer patients. Eur J Prev Cardiol.

[59] Bravo-Jaimes K, Palaskas NL, Banchs J, Abelhad NI, Altaf A, Gouni S (2021). Rate of progression of aortic stenosis in patients with cancer. Front Cardiovasc Med..

[60] Liang X, Wang Y, Yin X, Gong X, Pan S, Chen Z, Geng X (2020). Electrocardiographic characteristics of breast cancer patients treated with chemotherapy. Cardiol Res Pract.

[61] Khosravi N, Stoner L, Farajivafa V, Hanson ED (2019). Exercise training, circulating cytokine levels and immune function in cancer survivors: a meta-analysis. Brain Behav Immun.

[62] Lofterød T, Mortensen ES, Nalwoga H, Wilsgaard T, Frydenberg H, Risberg T (2018). Impact of pre-diagnostic triglycerides and HDL-cholesterol on breast cancer recurrence and survival by breast cancer subtypes. BMC Cancer.

[63] Buttros DDAB, Nahas EAP, Vespoli HDL, Uemura G, De Almeida BDR, Nahas-Neto J (2013). Risk of metabolic syndrome in postmenopausal breast cancer survivors. Menopause.

[64] Sutyagina AD, Shlyakhotka AV, Lyakhova EK, editors. Increasing the accuracy of oscillometric blood pressure measurement. In: 2018 IEEE Conference of Russian Young Researchers in Electrical and Electronic Engineering (EIConRus). IEEE; 2018.

[65] Acoltzin-Vidal C, Rabling-Arellanos EE, Marcial-Gallegos L (2010). Diagnóstico de la hipertensión arterial basado en el cálculo de la tensión arterial media. Inv Clín.

[66] Altini M, Van Hoof C, Amft O, editors. Relation between estimated cardiorespiratory fitness and running performance in free-living: an analysis of HRV4Training data. In: 2017 IEEE EMBS international conference on biomedical & health informatics (BHI). IEEE; 2017.

[67] Perrotta AS, Jeklin AT, Hives BA, Meanwell LE, Warburton DER (2017). Validity of the elite HRV smartphone application for examining heart rate variability in a field-based setting. J Strength Cond Res.

[68] Iwase T, Wang X, Shrimanker TV, Kolonin MG, Ueno NT (2021). Body composition and breast cancer risk and treatment: mechanisms and impact. Breast Cancer Res Treat.

[69] Hooshmand Moghadam B, Golestani F, Bagheri R, Cheraghloo N, Eskandari M, Wong A (2021). The effects of high-intensity interval training vs moderate-intensity continuous training on inflammatory markers, body composition, and physical fitness in overweight/obese survivors of breast cancer: a randomized controlled clinical trial. Cancers.

[70] McLester CN, Nickerson BS, Kliszczewicz BM, McLester JR (2020). Reliability and agreement of various InBody body composition analyzers as compared to dual-energy X-ray absorptiometry in healthy men and women. J Clin Densitom.

[71] Sturgeon KM, Mathis KM, Rogers CJ, Schmitz KH, Waning DL (2019). Cancer-and chemotherapy-induced musculoskeletal degradation. JBMR plus.

[72] Pescatello LS, Riebe D, Thompson PD (2014). ACSM's guidelines for exercise testing and prescription.

[73] Bruce RA (1974). Methods of exercise testing: step test, bicycle, treadmill, isometrics. Am J Cardiol.

[74] Karvonen MJ, Kentala E, Mustala O (1957). The effects of training on heart rate; a longitudinal study. Ann Med Exp Biol Fenn.

[75] Casla S, López-Tarruella S, Jerez Y, Marquez-Rodas I, Galvao DA, Newton RU (2015). Supervised physical exercise improves VO 2max, quality of life, and health in early stage breast cancer patients: a randomized controlled trial. Breast Cancer Res Treat.

[76] Rikli RE, Jones CJ. Senior fitness test manual: Human kinetics; 2013.

[77] Maréchal R, Fontvieille A, Parent-Roberge H, Fülöp T, Riesco E, Pavic M (2019). Effect of a mixed-exercise program on physical capacity and sedentary behavior in older adults during cancer treatments. Aging Clin Exp Res.

[78] Bail JR, Frugé AD, Cases MG, De Los Santos JF, Locher JL, Smith KP (2018). A home-based mentored vegetable gardening intervention demonstrates feasibility and improvements in physical activity and performance among breast cancer survivors. Cancer.

[79] Eston R, Evans HJL (2009). The validity of submaximal ratings of perceived exertion to predict one repetition maximum. J Sports Sci Med.

[80] Balsalobre-Fernández C, Geiser G, Krzyszkowski J, Kipp K (2020). Validity and reliability of a computer-vision-based smartphone app for measuring barbell trajectory during the snatch. J Sports Sci.

[81] Sañudo B, Rueda D, Pozo-Cruz BD, De Hoyo M, Carrasco L (2016). Validation of a video analysis software package for quantifying movement velocity in resistance exercises. J Strength Cond Res.

[82] Hales ME, Johnson BF, Johnson JT (2009). Kinematic analysis of the powerlifting style squat and the conventional deadlift during competition: is there a cross-over effect between lifts?. J Strength Cond Res.

[83] Boudreau SN, Dwyer MK, Mattacola CG, Lattermann C, Uhl TL, McKeon JM (2009). Hip-muscle activation during the lunge, single-leg squat, and step-up-and-over exercises. J Sport Rehabil.

[84] Santos WDND, Siqueira GDDJ, Martins WR, Vieira A, Schincaglia RM, Gentil P (2019). Reliability and agreement of the 10-repetition maximum test in breast cancer survivors. Front Oncol.

[85] Hernández-Belmonte A, Martínez-Cava A, Morán-Navarro R, Courel-Ibáñez J, Pallarés J (2021). A comprehensive analysis of the velocity-based method in the shoulder press exercise: stability of the load-velocity relationship and sticking region parameters. Biol Sport.

[86] Fernandes JF, Lamb KL, Twist C (2018). A comparison of load-velocity and load-power relationships between well-trained young and middle-aged males during three popular resistance exercises. J Strength Cond Res.

[87] Moreira OC, Faraci LL, de Matos DG, Mazini Filho ML, Da Silva SF, Aidar FJ (2017). Cardiovascular responses to unilateral, bilateral, and alternating limb resistance exercise performed using different body segments. J Strength Cond Res.

[88] Nikander R, Sievänen H, Ojala K, Oivanen T, Kellokumpu-Lehtinen P-L, Saarto T (2007). Effect of a vigorous aerobic regimen on physical performance in breast cancer patients—a randomized controlled pilot trial. Acta Oncol.

[89] Jiménez-Reyes P, Samozino P, Brughelli M, Morin J-B (2017). Effectiveness of an individualized training based on force-velocity profiling during jumping. Front Physiol.

[90] Jiménez-Reyes P, Samozino P, Cuadrado-Peñafiel V, Conceição F, González-Badillo JJ, Morin J-B (2014). Effect of countermovement on power–force–velocity profile. Eur J Appl Physiol.

[91] Balsalobre-Fernández C, Glaister M, Lockey RA (2015). The validity and reliability of an iPhone app for measuring vertical jump performance. J Sports Sci.

[92] Jiménez-Reyes P, Samozino P, Pareja-Blanco F, Conceição F, Cuadrado-Peñafiel V, González-Badillo JJ (2017). Validity of a simple method for measuring force-velocity-power profile in countermovement jump. Int J Sports Physiol Perform.

[93] Jiménez-Reyes P, Samozino P, García-Ramos A, Cuadrado-Peñafiel V, Brughelli M, Morin J-B (2018). Relationship between vertical and horizontal force-velocity-power profiles in various sports and levels of practice. PeerJ.

[94] Romero-Franco N, Jiménez-Reyes P, Castaño-Zambudio A, Capelo-Ramírez F, Rodríguez-Juan JJ, González-Hernández J (2017). Sprint performance and mechanical outputs computed with an iPhone app: comparison with existing reference methods. Eur J Sport Sci.

[95] Foley MP, Hasson SM (2016). Effects of a community-based multimodal exercise program on health-related physical fitness and physical function in breast cancer survivors: a pilot study. Integr Cancer Ther.

[96] Suni J, Husu P, Rinne M. Fitness for health: the ALPHA-FIT test battery for adults aged 18–69. Tester’s Manual Tampare, Finland: Published by European Union DS, and the UKK Institute for Health Promotion Research; 2009.

[97] Arraras J, Arias F, Tejedor M, Pruja E, Marcos M, Martínez E (2002). The EORTC QLQ-C30 (version 3.0) quality of life questionnaire: validation study for Spain with head and neck cancer patients. Psycho-Oncol J Psychol Soc Behav Dimens Cancer..

[98] Fayers P, Aaronson NK, Bjordal K, Sullivan M. EORTC QLQ–C30 scoring manual: European Organisation for Research and Treatment of Cancer; 1995.

[99] Webster K, Cella D, Yost K (2003). The F unctional A ssessment of C hronic I llness T herapy (FACIT) Measurement System: properties, applications, and interpretation. Health Qual Life Outcomes.

[100] Yellen SB, Cella DF, Webster K, Blendowski C, Kaplan E (1997). Measuring fatigue and other anemia-related symptoms with the Functional Assessment of Cancer Therapy (FACT) measurement system. J Pain Symptom Manag.

[101] Diener E, Emmons R, Larsen R, Griffin S (1985). The life satisfaction scale. J Pers Assess.

[102] Cerezo MV, Soria-Reyes LM, Alarcón R, Blanca MJ (2022). The Satisfaction with Life Scale in breast cancer patients: Psychometric properties. Int J Clin Health Psychol.

[103] Rosenberg M (2015). Society and the adolescent self-image.

[104] Cobo-Cuenca AI, Martín-Espinosa NM, Rodríguez-Borrego MA, Carmona-Torres JM (2019). Determinants of satisfaction with life and self-esteem in women with breast cancer. Qual Life Res.

[105] Vázquez-Morejón Jiménez R, Jiménez García-Bóveda R, Vázquez Morejón AJ (2004). Escala de autoestima de Rosenberg: fiabilidad y validez en población clínica española. Apunt Psicol.

[106] Zigmond AS, Snaith RP (1983). The hospital anxiety and depression scale. Acta Psychiatr Scand.

[107] López-Roig S, Terol M, Pastor M, Neipp M, Massutí B (2000). Ansiedad y depression. Validación de la escala HAD en pacientes oncológicos. Rev Psicol Salud..

[108] Gummesson C, Ward MM, Atroshi I (2006). The shortened disabilities of the arm, shoulder and hand questionnaire (Quick DASH): validity and reliability based on responses within the full-length DASH. BMC Musculoskelet Disord.

[109] LeBlanc M, Stineman M, DeMichele A, Stricker C, Mao JJ (2014). Validation of QuickDASH outcome measure in breast cancer survivors for upper extremity disability. Arch Phys Med Rehabil.

[110] Gómez-Pérez L, López-Martínez AE, Ruiz-Párraga GT (2011). Psychometric properties of the Spanish version of the Tampa Scale for Kinesiophobia (TSK). J Pain.

[111] Tkachuk GA, Harris CA (2012). Psychometric properties of the Tampa Scale for Kinesiophobia-11 (TSK-11). J Pain.

[112] Ruiz-Casado A, Alejo LB, Santos-Lozano A, Soria A, Ortega MJ, Pagola I (2016). Validity of the physical activity questionnaires IPAQ-SF and GPAQ for cancer survivors: insights from a Spanish cohort. Int J Sports Med.

[113] Murcia J, Gimeno EC, Camacho AM (2007). Measuring self-determination motivation in a physical fitness setting: validation of the Behavioural Regulation in Exercise Questionnaire-2 (BREQ-2) in a Spanish sample. J Sport Med Phys Fit.

[114] Cid L, Monteiro D, Teixeira D, Teques P, Alves S, Moutão J (2018). The behavioral regulation in exercise questionnaire (BREQ-3) Portuguese-version: evidence of reliability, validity and invariance across gender. Front Psychol.

[115] Frey F. SPSS (software). The International Encyclopedia of Communication Research Methods. 2017. p. 1–2.

[116] Lavín-Pérez AM, Collado-Mateo D, Mayo X, Liguori G, Humphreys L, Jiménez A (2021). Can exercise reduce the autonomic dysfunction of patients with cancer and its survivors? a systematic review and meta-analysis. Front Psychol.

[117] JB SG, Silva-Filho A, Dias C, Leite R, Mostarda C (2017). Effect of exercise training and detraining in autonomic modulation and cardiorespiratory fitness in breast cancer survivors. J Sports Med Phys Fit.

[118] Mostarda C, Castro-Filha J, Reis AD, Sevílio M, Dias CJ, Silva-Filho AC (2017). Short-term combined exercise training improves cardiorespiratory fitness and autonomic modulation in cancer patients receiving adjuvant therapy. J Exerc Rehabil.

[119] Niederer D, Vogt L, Thiel C, Schmidt K, Bernhörster M, Lungwitz A (2013). Exercise effects on HRV in cancer patients. Int J Sports Med.

[120] Shin H-C, Yang J-O, Kim S-R (2016). Effects of circuit exercise on autonomic nerve system of survivors after surgery of breast cancer. J Phys Ther Sci.

[121] Caro-Moran E, Fernandez-Lao C, Galiano-Castillo N, Cantarero-Villanueva I, Arroyo-Morales M, Díaz-Rodríguez L (2016). Heart rate variability in breast cancer survivors after the first year of treatments: a case-controlled study. Biol Res Nurs.

[122] Arab C, Dias DPM, de Almeida Barbosa RT, de Carvalho TD, Valenti VE, Crocetta TB (2016). Heart rate variability measure in breast cancer patients and survivors: a systematic review. Psychoneuroendocrinology.

[123] Palma MR, Tebar WR, Vanderlei LCM, Fregonesi CEPT, Ribeiro FE, Caldeira DT, Ritti-Dias RM, Christofaro DGD (2022). Association between cardiac autonomic modulation and sedentary behavior in breast cancer survivors: a 12-month cohort study. Support Care Cancer.

[124] Scott JM, Jones LW, Hornsby WE, Koelwyn GJ, Khouri MG, Joy AA (2014). Cancer therapy-induced autonomic dysfunction in early breast cancer: implications for aerobic exercise training. Int J Cardiol.

[125] Kingwell BA (2000). Nitric oxide as a metabolic regulator during exercise: effects of training in health and disease. Clin Exp Pharmacol Physiol.

[126] Routledge FS, Campbell TS, McFetridge-Durdle JA, Bacon SL (2010). Improvements in heart rate variability with exercise therapy. Can J Cardiol.

[127] Plews DJ, Laursen PB, Le Meur Y, Hausswirth C, Kilding AE, Buchheit M (2014). Monitoring training with heart rate-variability: how much compliance is needed for valid assessment?. Int J Sports Physiol Perform.

[128] Antunes P, Esteves D, Nunes C, Sampaio F, Ascensão A, Vilela E (2019). Impact of exercise training on cardiotoxicity and cardiac health outcomes in women with breast cancer anthracycline chemotherapy: a study protocol for a randomized controlled trial. Trials.

[129] de Sá Feitosa LA, dos Santos CJ, Dantas CO, de Souza DS, de Vasconcelos CML, Miguel-dos-Santos R (2021). Resistance training improves cardiac function and cardiovascular autonomic control in doxorubicin-induced cardiotoxicity. Cardiovasc Toxicol.

[130] Pfannenstiel K, Hayward R (2018). Effects of resistance exercise training on doxorubicin-induced cardiotoxicity. J Cardiovasc Pharmacol.

[131] Murray J, Bennett H, Bezak E, Perry R (2022). The role of exercise in the prevention of cancer therapy-related cardiac dysfunction in breast cancer patients undergoing chemotherapy: systematic review. Eur J Prev Cardiol.

[132] Collado-Mateo D, Lavín-Pérez AM, Peñacoba C, Del Coso J, Leyton-Román M, Luque-Casado A (2021). Key factors associated with adherence to physical exercise in patients with chronic diseases and older adults: an umbrella review. Int J Environ Res Public Health.

[133] Grazioli E, Cerulli C, Dimauro I, Moretti E, Murri A, Parisi A (2020). New strategy of home-based exercise during pandemic COVID-19 in breast cancer patients: a case study. Sustainability.

[134] Natalucci V, Marini CF, Flori M, Pietropaolo F, Lucertini F, Annibalini G (2021). Effects of a home-based lifestyle intervention program on cardiometabolic health in breast cancer survivors during the COVID-19 lockdown. J Clin Med.

[135] Yuan Y, Lin L, Zhang N, Xie C, Liang J, Qi Y, Dong B, Tian L (2022). Effects of home-based walking on cancer-related fatigue in patients with breast cancer: a meta-analysis of randomized controlled trials. Arch Phys Med Rehabil.

[136] Coughlin SS, Caplan LS, Williams V (2019). Home-based physical activity interventions for breast cancer patients receiving primary therapy: a systematic review. Breast Cancer Res Treat.

[137] Huizinga F, Westerink N, Berendsen AJ, Walenkamp AM, Oude JN, Berger M (2021). Home-based physical activity to alleviate fatigue in cancer survivors: a systematic review and meta-analysis. Med Sci Sports Exerc.

[138] Ochi E, Tsuji K, Narisawa T, Shimizu Y, Kuchiba A, Suto A, Jimbo K, Takayama S, Ueno T, Sakurai N, Matsuoka Y (2022). Cardiorespiratory fitness in breast cancer survivors: a randomised controlled trial of home-based smartphone supported high intensity interval training. BMJ Support Palliat Care.

